# Supplementary material for: Collateral sensitivity to pleuromutilins in vancomycin-resistant Enterococcus faecium
Source: Nat Commun. 2022 Apr 7;13:1888. doi: 10.1038/s41467-022-29493-0 (PMC8990069; doi:10.1038/s41467-022-29493-0)
Supplement: Supplementary file 1 — supplementary information [file 41467_2022_29493_MOESM1_ESM.pdf]

Supplementary for

Collateral sensitivity to pleuromutilins in vancomycin-resistant *Enterococcus faecium*

Qian Li[†], Shang Chen[†], Kui Zhu[*], Yucheng Huang, Zhangqi Shen, Shuangyang Ding, Danxia Gu, Qiwen Yang, Hong-li Sun, Fupin Hu, Hui Wang, Jiachang Cai, Bing Ma, Rong Zhang[*], Jianzhong Shen[*]

† These authors contributed equally to this work.
* Corresponding authors.
Email: zhuk@cau.edu.cn (K.Z.); zhang-rong@zju.edu.cn (R.Z.); sjz@cau.edu.cn (J.S.)

This file includes:

Supplementary Figs. 1 to 22
Supplementary Tables 1 to 8
Reference

Content list

Supplementary Figures

Supplementary Fig. 1 -- 1
Supplementary Fig. 2 -- 2
Supplementary Fig. 3 -- 3
Supplementary Fig. 4 -- 4
Supplementary Fig. 5 -- 5
Supplementary Fig. 6 -- 6
Supplementary Fig. 7 -- 7
Supplementary Fig. 8 -- 8
Supplementary Fig. 9 -- 9
Supplementary Fig. 10 -- 10
Supplementary Fig. 11 -- 11
Supplementary Fig. 12 -- 12
Supplementary Fig. 13 -- 13
Supplementary Fig. 14 -- 14
Supplementary Fig. 15 -- 15
Supplementary Fig. 16 -- 16
Supplementary Fig. 17 -- 17
Supplementary Fig. 18 -- 18
Supplementary Fig. 19 -- 19
Supplementary Fig. 20 -- 20
Supplementary Fig. 21 -- 21
Supplementary Fig. 22 -- 22

Supplementary Tables

Supplementary Table 1 -- 23
Supplementary Table 2 -- 24
Supplementary Table 3 -- 25
Supplementary Table 4 -- 26
Supplementary Table 5 -- 27
Supplementary Table 6 -- 28
Supplementary Table 7 -- 29
Supplementary Table 8 -- 30

Reference -- 31

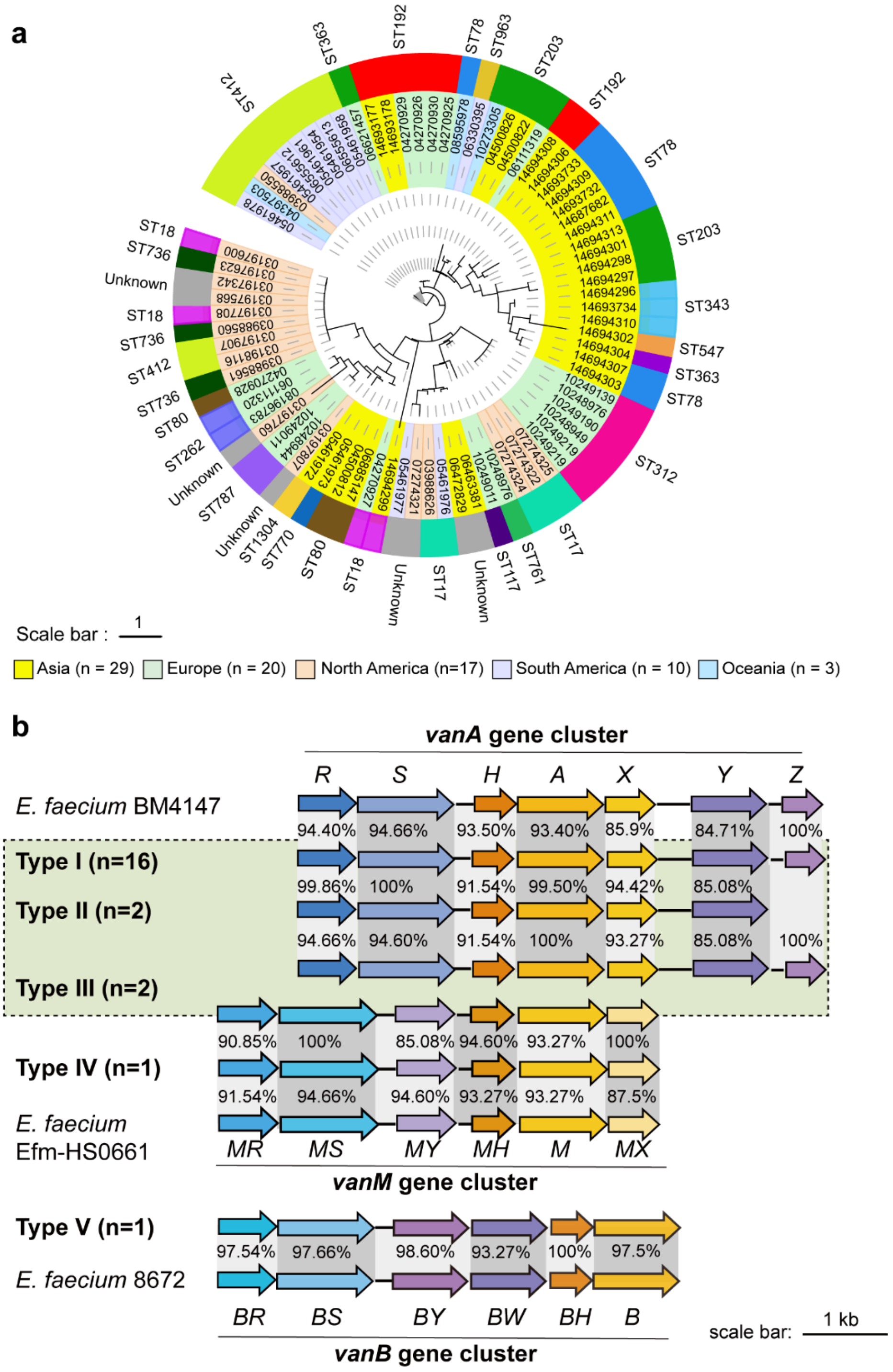

Supplementary Fig. 1 Phylogenetic tree of VRE$_{fm}$ genomes and types of *van* gene cluster

(**a**) Phylogenetic tree of prevalent *van* gene clusters in global VRE$_{fm}$ isolates, including *vanA* (n = 71), *vanB* (n = 7) and *vanM* (n = 1). Color strips of the outer ring show the MLST types. More details of the isolates can be found in Supplementary Table 3.

(**b**) Genotypes of *van* gene clusters in 22 clinical VRE$_{fm}$ isolates. *E. faecium* BM4147, *E. faecium* Efm-HS0661 and *E. faecium* 8672 are reference strains for *vanA*, *vanM* and *vanB*, respectively.

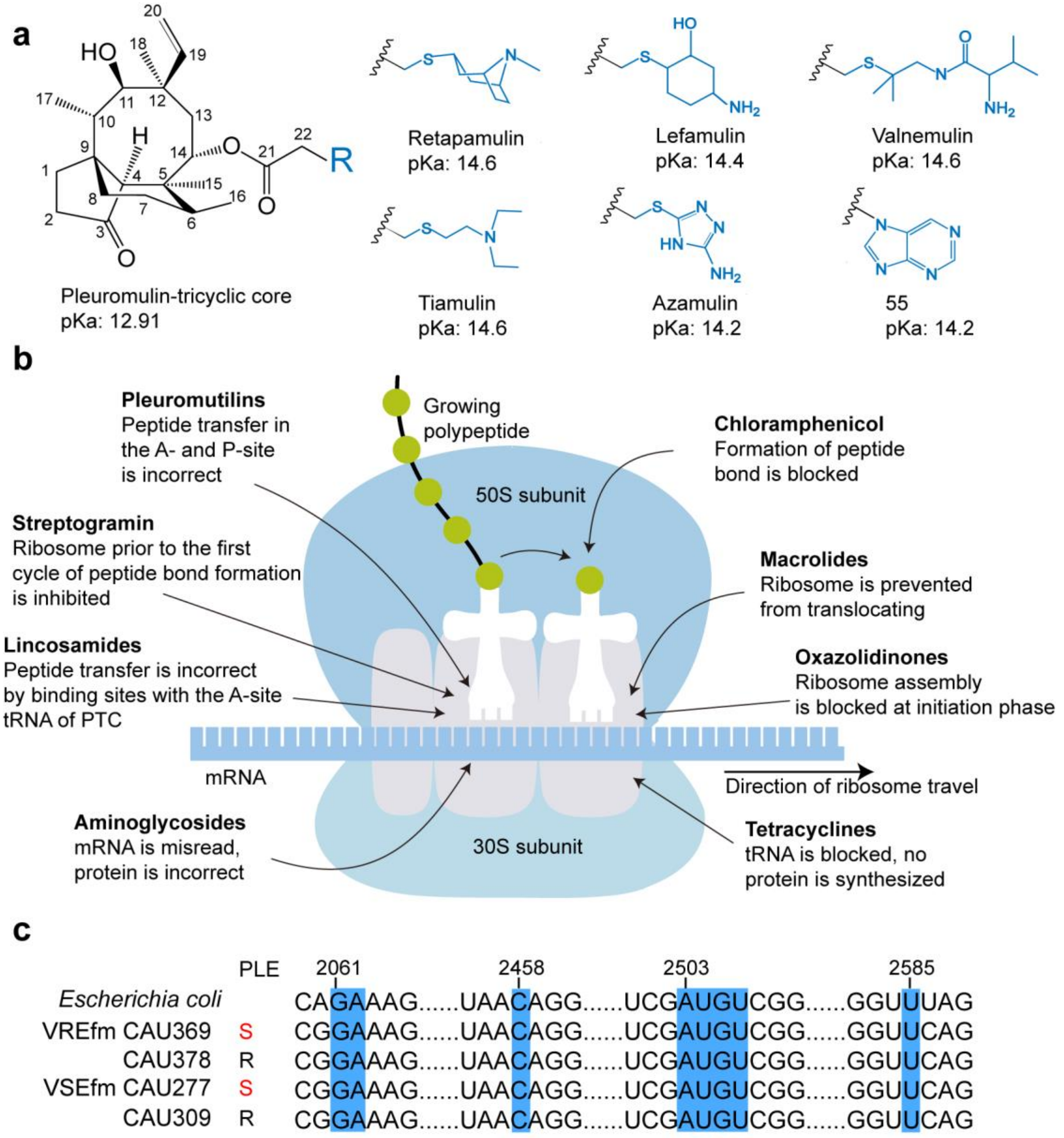

Supplementary Fig. 2 The mode of action of pleuromutilins

(**a**) Chemical structures of pleuromutilins. Compound **55** in (**a**) was named in the reference[1].

(**b**) Modes of action of ribosome-targeting antibiotics.

(**c**) Binding sites of pleuromutilins in the PTC domain. No mutations were found at the sites in 40 *E. faeciums* isolates. More details about the binding sites were shown in Supplementary Table 4.

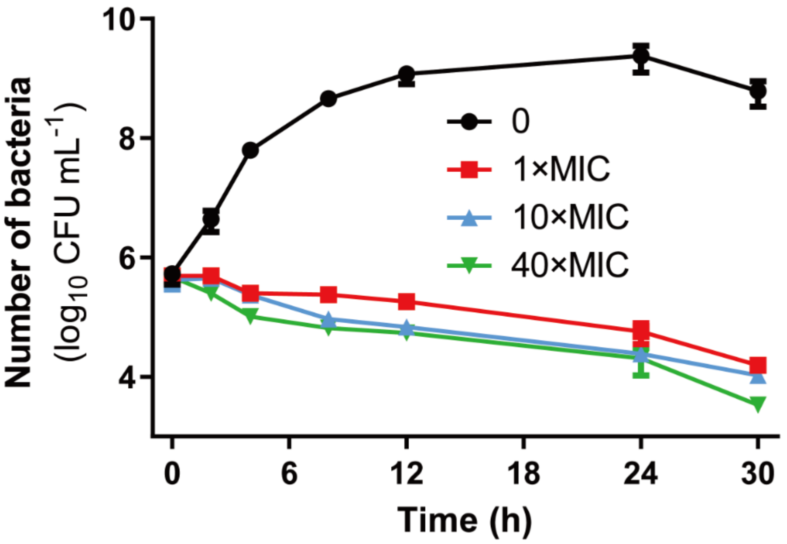

Supplementary Fig. 3 Bacteriostatic activity of lefamulin against VRE$_{fm}$.

Time-dependent killing curves of VRE$_{fm}$ CAU369 in the presence of lefamulin. VRE$_{fm}$ CAU369 at exponential phase were challenged with 1×MIC, 10×MIC and 40×MIC lefamulin. Experiments were performed as three biologically independent experiments, and the mean ± S.D. (n = 3) is shown. *P* values were determined by non-parametric one-way ANOVA.

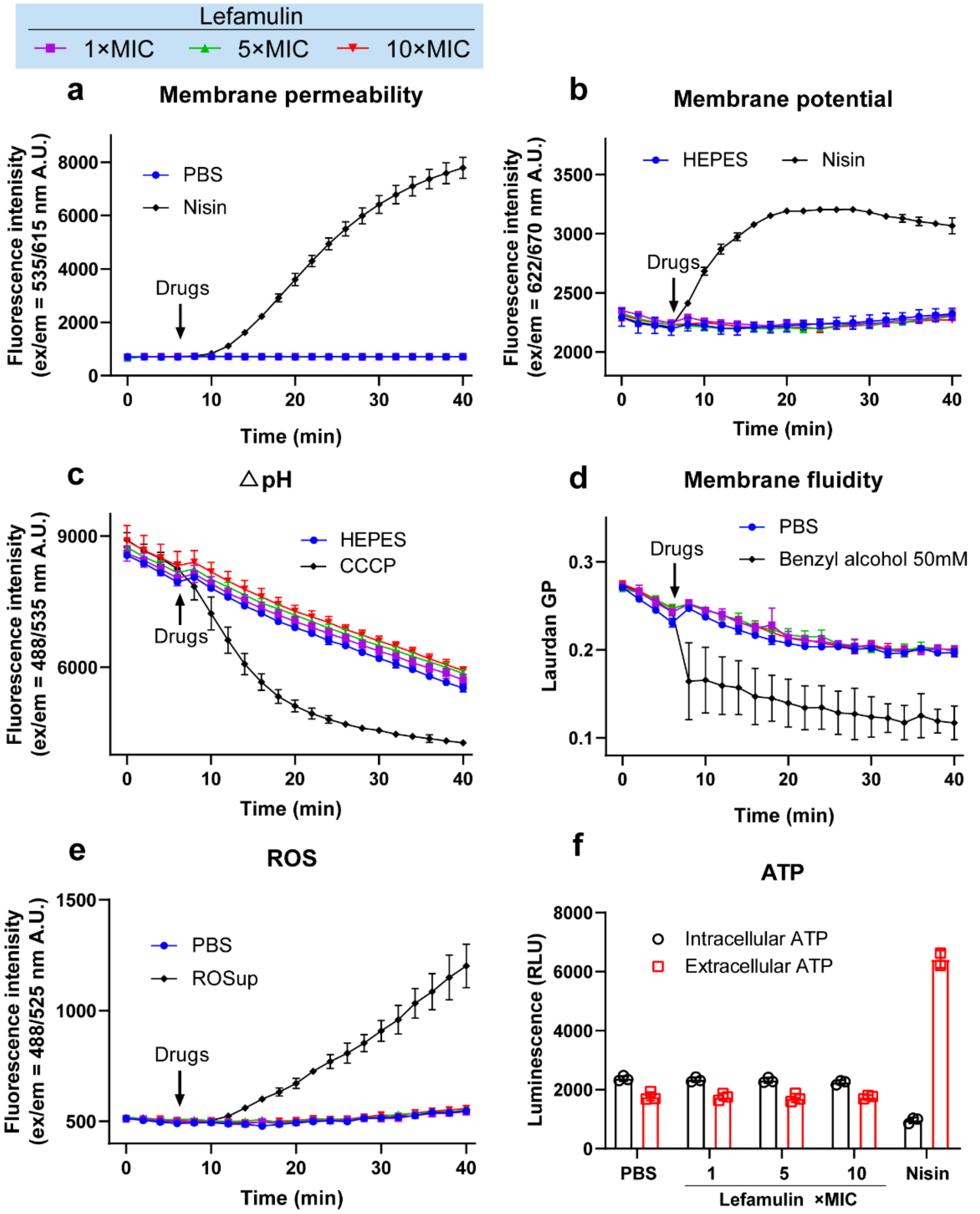

Supplementary Fig. 4 Maintained integrity and functions of VRE$_{fm}$ membrane. (**a-d**) Membrane permeability (**a**), membrane potential (**b**), △pH (**c**) and membrane fluidity (**d**) of VRE$_{fm}$ CAU369 treated with lefamulin at the levels of 1×MIC, 5×MIC and 10×MIC. The arrows indicate the time points of added compounds. Nisin (100 µg/mL), CCCP (50 µM) and benzyl alcohol (50 mM) were used as positive controls. PI (10 µM), DiSC3(5) (1 µM), BCECF-AM (10 µM) and Laurdan were used as probes.

(**e-f**) ROS accumulation (**e**) and levels of intracellular and extracellular ATP (**f**) in VRE$_{fm}$ CAU369 in the presence of lefamulin. ROSup and Nisin (100 µg/mL) were used as positive controls.

Experiments were performed as three biologically independent experiments, and the mean ± S.D. (n = 3) is shown. *P* values were determined by non-parametric one-way ANOVA.

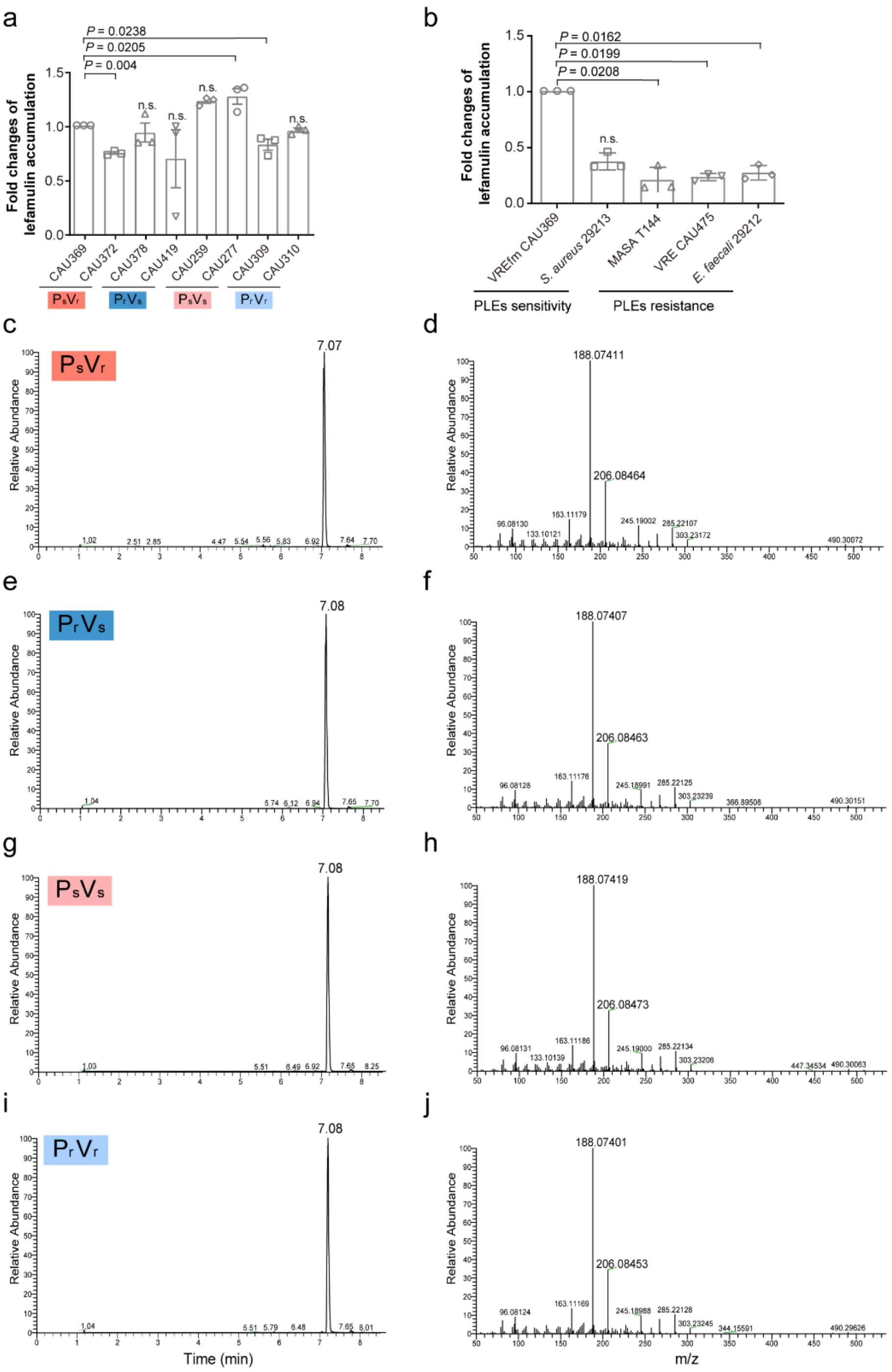

Supplementary Fig. 5 Accumulated lefamulin in bacteria.

(**a-b**) Fold changes of lefamulin accumulation in diverse isolates of *E. faeciums* (**a**), *E. faecalis* and *S. aureus* (**b**). Accumulations of lefamulin in *E. faeciums* (n = 8), *E. faecalis* (n = 2) and *S. aureus* (n = 2) after treatments of 0.3 µg/mL lefamulin for 1 h. (**c-j**) Chromatographic and MS/MS spectra of lefamulin in four phenotypes of *E. faeciums* isolates. No further chemical modifications were observed in all isolates. Experiments were performed as three biologically independent experiments, and the mean ± S.D. (n = 3) is shown. *P* values were determined by non-parametric one-way ANOVA.

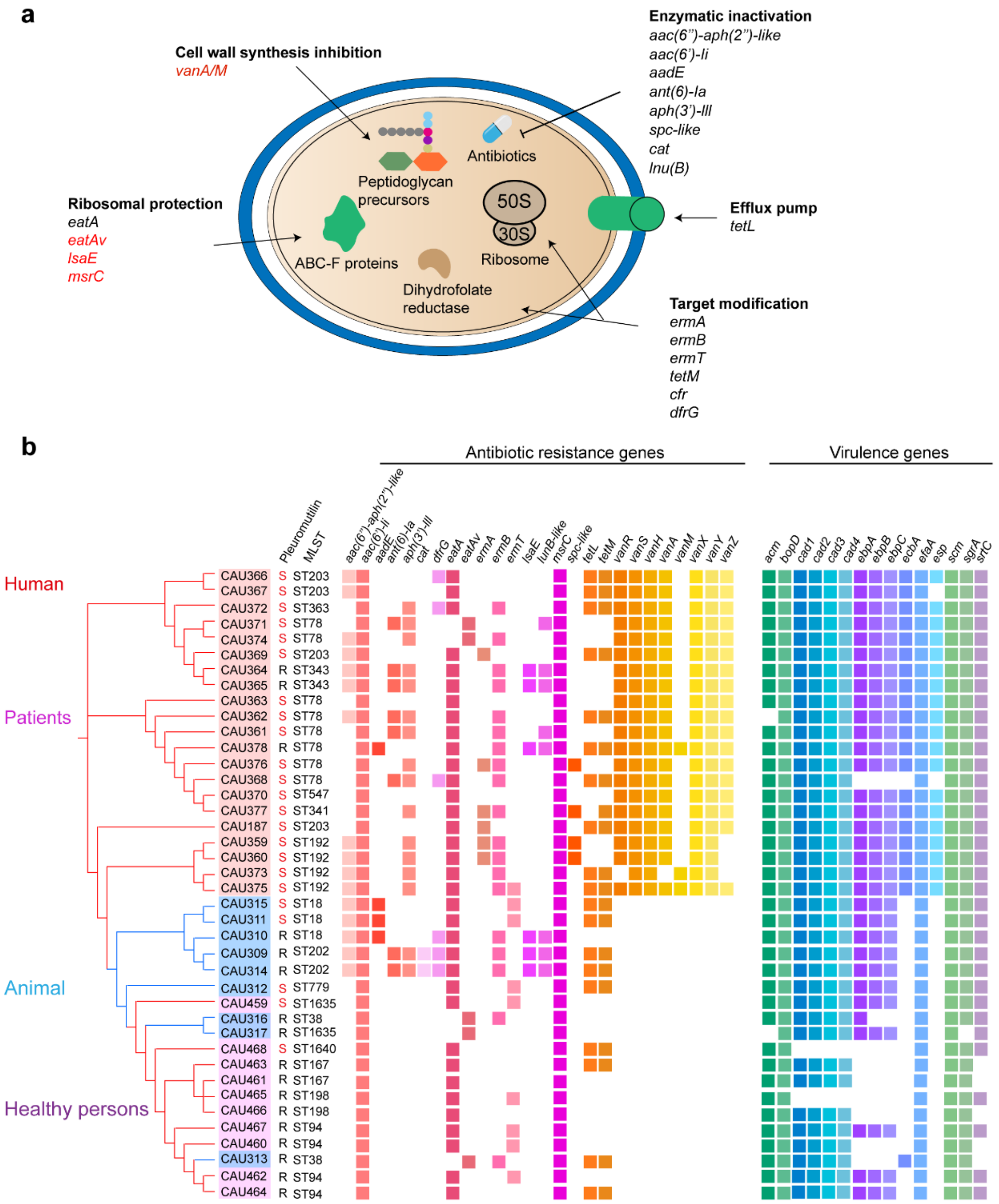

Supplementary Fig. 6 Multiple resistance genes and virulence genes in *E. faeciums* isolates

(**a**) Scheme of mechanisms of resistance to antibiotics in *E. faecium*.

(**b**) Multiple resistance genes and virulence genes in 40 *E. faeciums* isolates. Phylogenetic tree of 40 *E. faeciums* isolates of different origins was shown (Left). Red "S" represents that isolates are sensitive to pleuromutilins, and black "R" represents resistance. No *cfr* was found in these isolates.

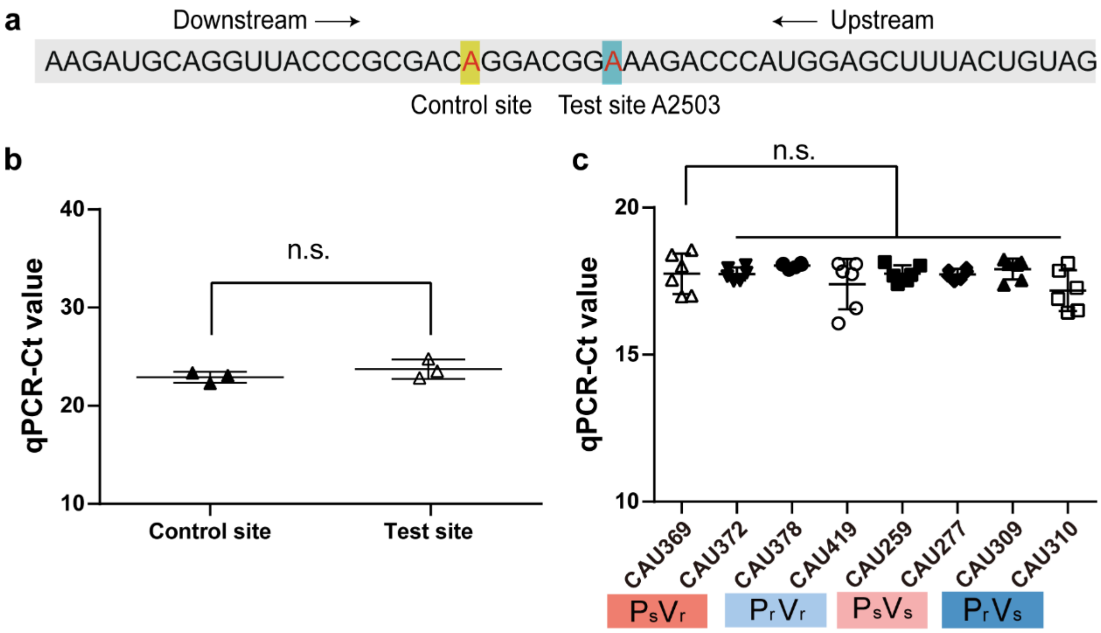

Supplementary Fig. 7 No methylation on A2503 in *E. faecium*.

(**a**) Locations of A2503 and control site in sequence of PTC.
(**b**) qPCR-Ct values of select m^6A of control site and test site in VRE_{fm} CAU369. Control site was used as non-methylated negative controls compared with test site. Test site is not methylated. All data are presented as mean ± S.D (n = 3).
(**c**) qPCR-Ct values of select m^6A of test sites in four phenotypic isolates. A2503 site in VRE_{fm} CAU369 was used as non-methylated negative controls compared to other isolates. All data are presented as mean ± S.D (n = 6).
P values were determined by non-parametric one-way ANOVA.

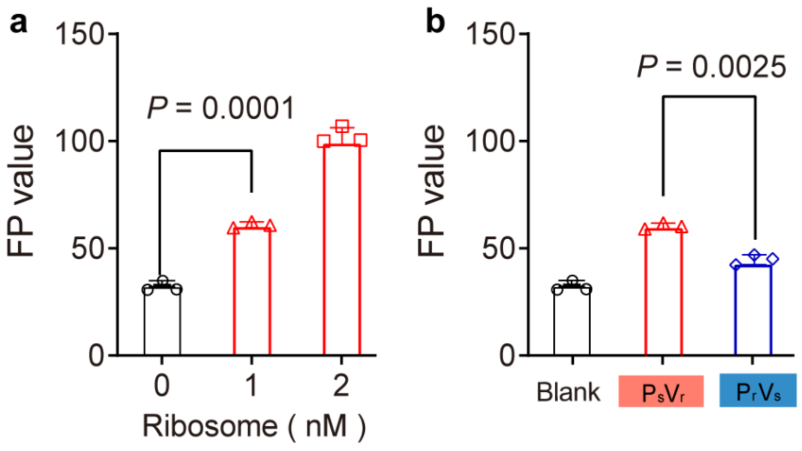

Supplementary Fig. 8 Increased binding of pleuromutilins to ribosomes in P_sV_r VRE$_{fm}$.

(**a**) The fluorescence polarization values (FP value) between ribosomes of VRE$_{fm}$ CAU369 and pleuromutilin-tracers (using 0.5 μM VAL-DTAF as a model).
(**b**) FP values of 0.5 μM pleuromutilin-tracers binding to the ribosomes (1 nM) of VRE$_{fm}$ CAU369 or VSE$_{fm}$ CAU310.
Experiments were performed as three biologically independent experiments, and the mean ± S.D. (n = 3) is shown. *P* values were determined by non-parametric one-way ANOVA.

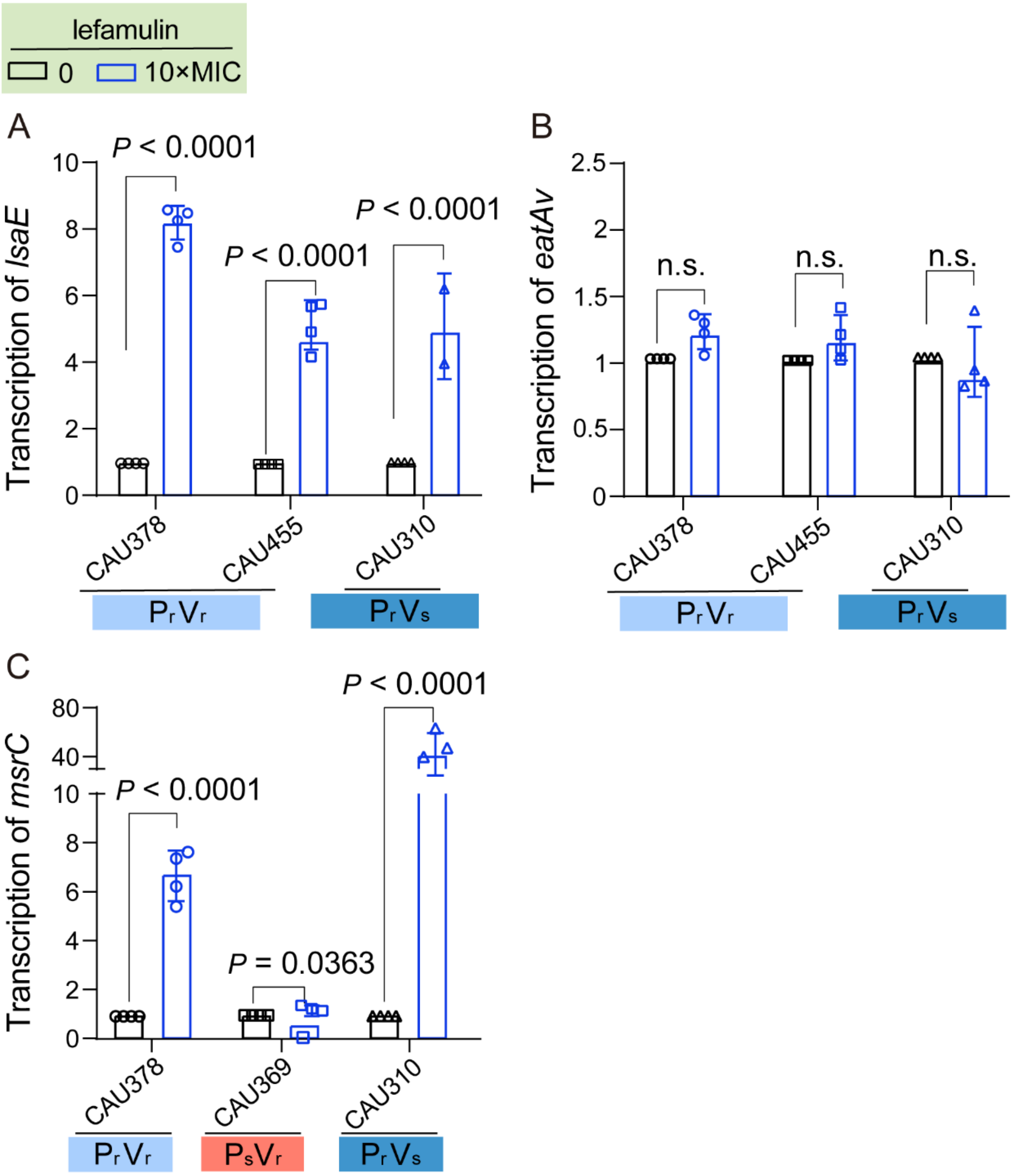

Supplementary Fig. 9 Collateral sensitivity in P_SV_R VRE$_{fm}$ with decreased *msrC* transcription.

(**a**-**c**) Transcription analysis of *lsaE* (**a**), *eatAv* (**b**) and *msrC* (**c**).
E. faeciums isolates were treated with lefamulin (0.3 µg/mL) for 1 h.
Experiments were performed as three biologically independent experiments, and the mean ± S.D. (n = 4) is shown. *P* values were determined by non-parametric one-way ANOVA.

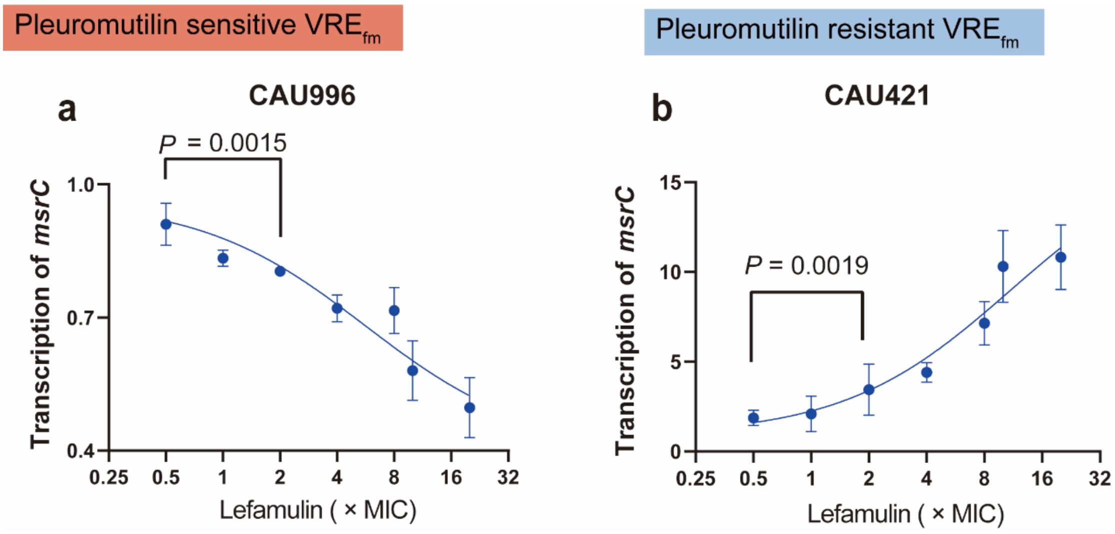

Supplementary Fig. 10 Transcription of *msrC* in VRE$_{fm}$ in the presense of lefamulin. Transcription analysis of *msrC* in the pleuromutilin sensitive isolate VRE$_{fm}$ CAU996 (**a**) and the resistant isolate VRE$_{fm}$ CAU421 (**b**). Both VRE$_{fm}$ isolates were treated with lefamulin for 1 h. Experiments were performed as three biologically independent experiments, and the mean ± S.D. (n = 3) were shown. *P* values were determined by non-parametric one-way ANOVA.

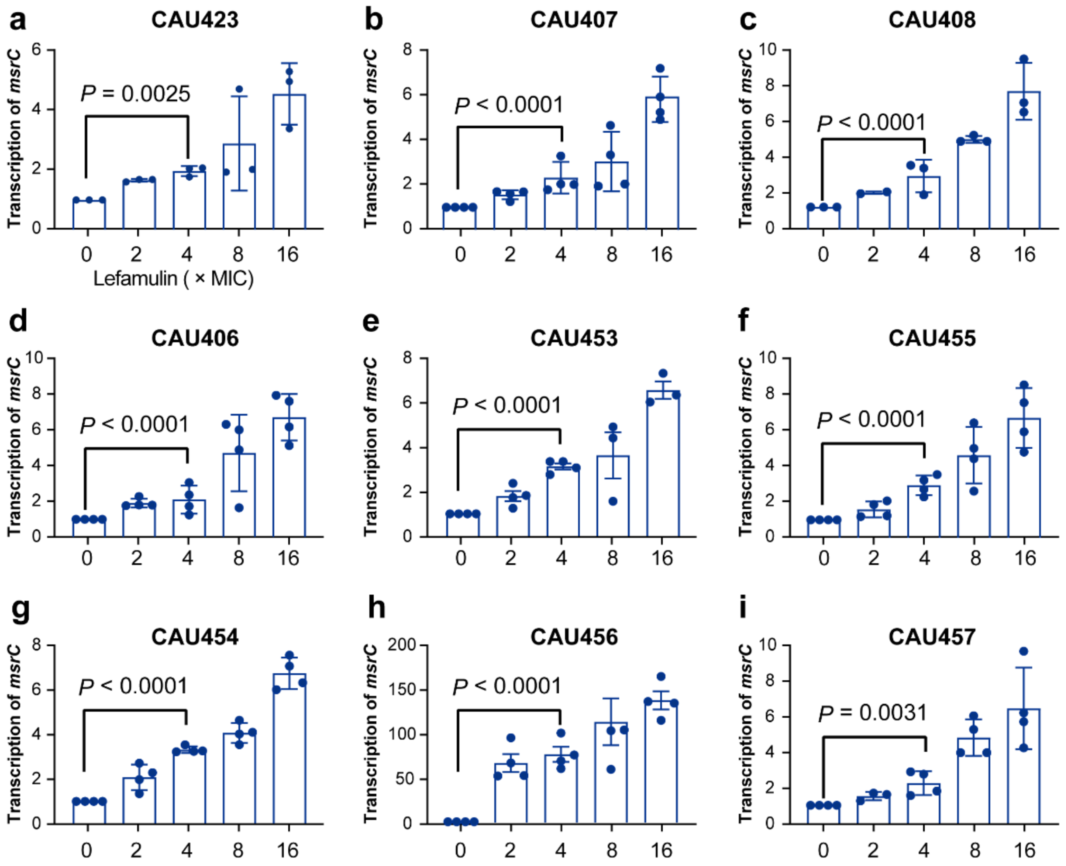

Supplementary Fig. 11 Transcription of *msrC* in pleuromutilin resistant VRE_{fm} isolates increased in a dose-dependent manner.

Transcription analysis of *msrC* in pleuromutilin resistant VRE_{fm} isolates (**a-i**). VRE_{fm} isolates were treated with lefamulin for 1 h. Experiments were performed as three biologically independent experiments, and the mean ± S.D. (n = 3) is shown. *P* values were determined by non-parametric one-way ANOVA.

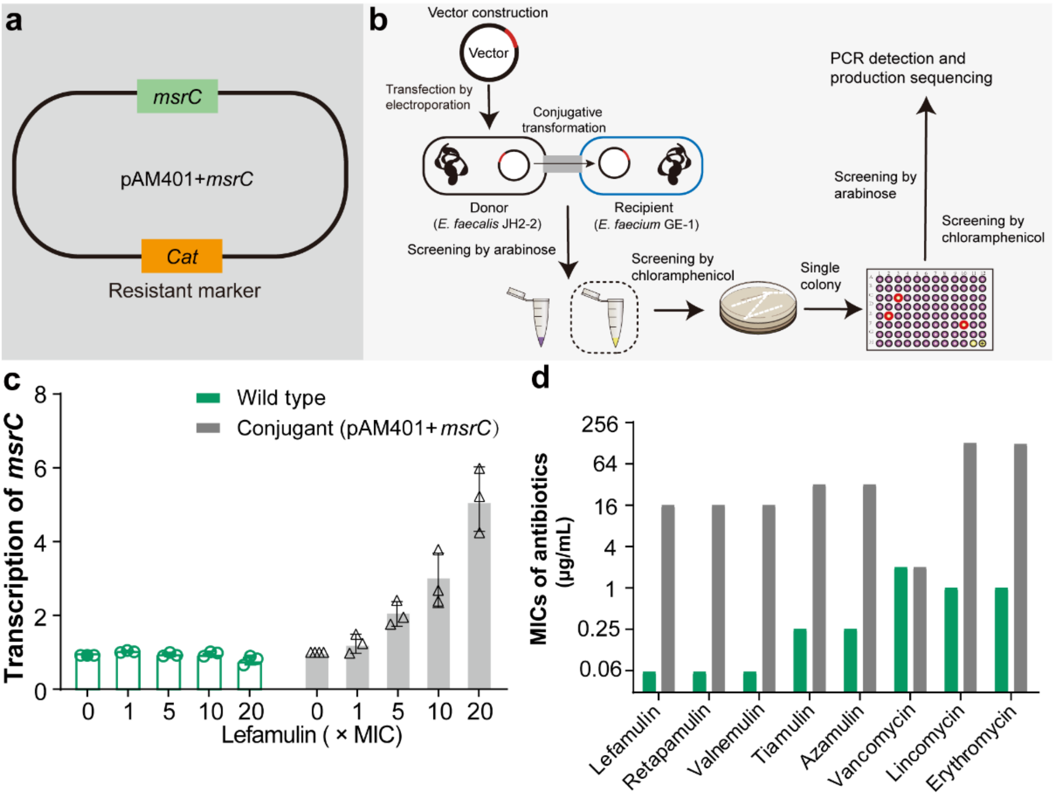

Supplementary Fig. 12 The overexpression of *msrC* induces pleuromutilins resistance.

(**a-b**) Design of *msrC* expression plasmid (**a**) and scheme of transformation (**b**).

(**c**) The expression of *msrC* in the mutant (pAM401+ *msrC*) in the presence of a concentration gradient of lefamulin. Experiments were performed as three biologically independent experiments, and the mean ± S.D. (n = 3) is shown.

(**d**) Comparison of antibiotic susceptibility in wild-type *E. faecium* and mutant.

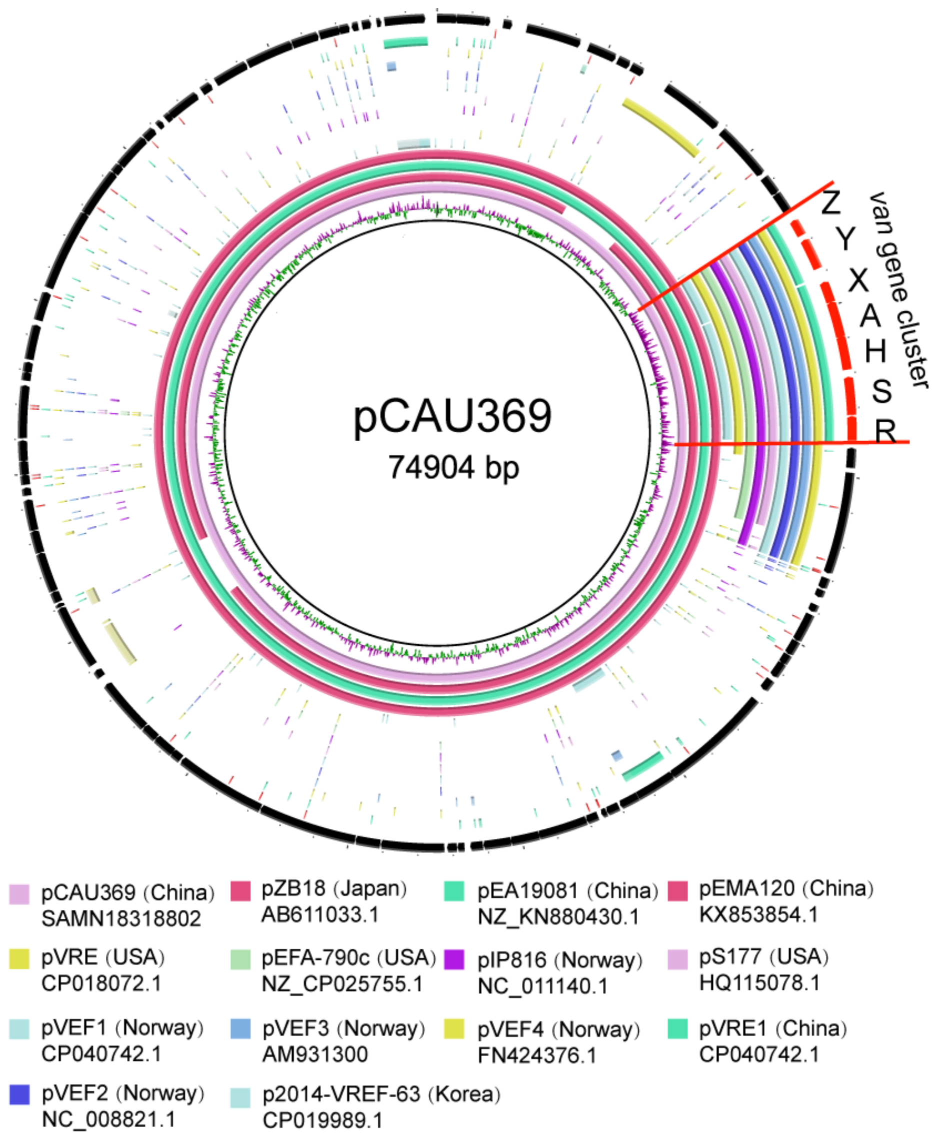

Supplementary Fig. 13 The *vanA* gene clusters in global plasmids.

Locations of the *vanA* gene clusters are shown in 14 plasmids. The plasmid pCAU369 in VRE_{fm} CAU369 shares the same sequence of the *vanA* gene cluster with that in the other 13 global plasmids. The identities of the *vanA* genes are 100%.

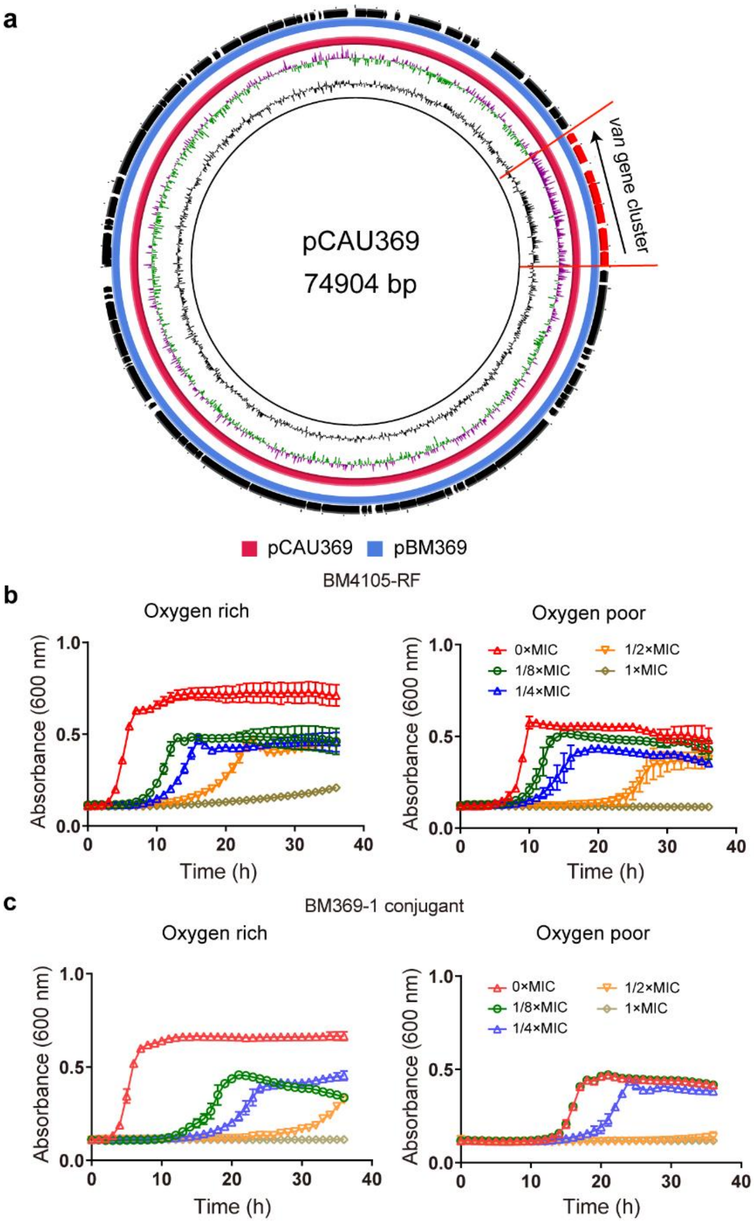

Supplementary Fig. 14 Characterization of the conjugant with the plasmid pCAU369.

(**a**) High similarity between the plasmid pCAU369 and pBM369 in the conjugant. pCAU369 is from VRE CAU369 (donor), pBM369 is from the conjugant BM369-1. (**b**) Growth dynamics of *E. faeciums* BM4105-RF (receptor) treated with sublethal levels of lefamulin under oxygen rich (left) and poor (right) conditions. (**c**) Growth dynamics of the conjugant *E. faeciums* BM369-1 of *E. faeciums* BM4105-RF treated with sublethal levels of lefamulin under oxygen rich (left) and poor (right) conditions.

Experiments were performed as three biologically independent experiments, and the mean ± S.D. (n = 3) is shown. *P* values were determined by non-parametric one-way ANOVA.

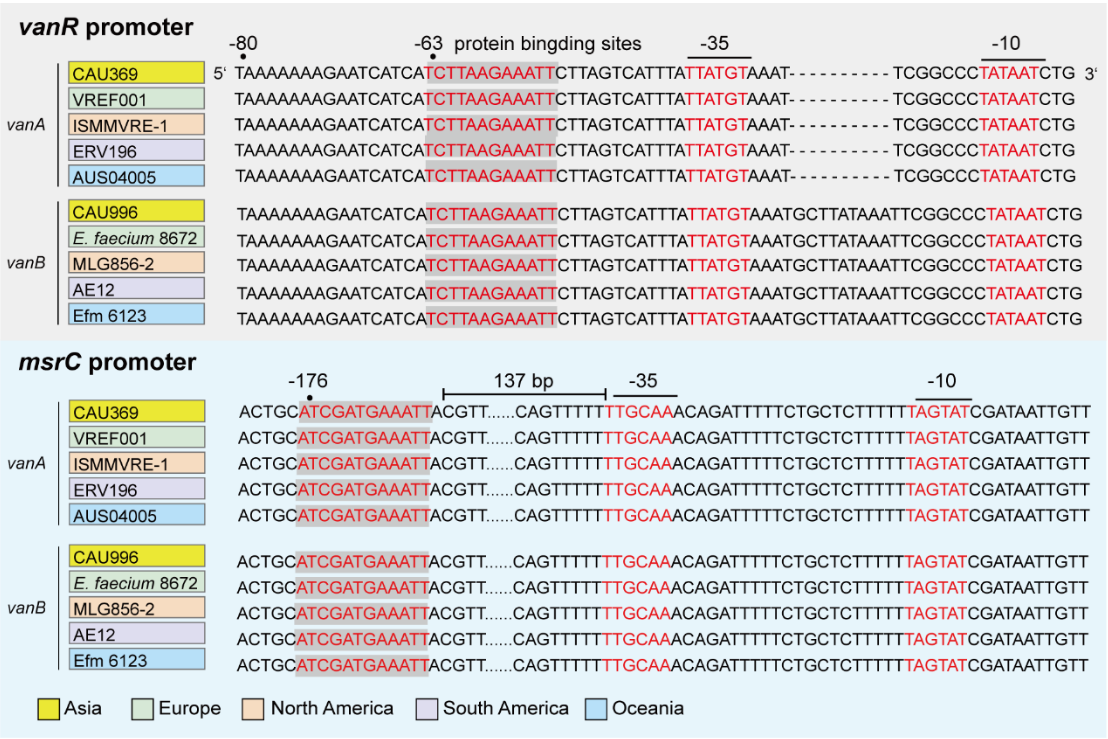

Supplementary Fig. 15 The *vanA* and *vanB* type *E. faecium* share the same patterns in the promoters of *msrC/vanR*. CAU369, VREF001, ISMMVRE-1, ERV196, and AUS04005 are *vanA* type isolates; CAU996, *E. faecium* 8672, MLG856-2, AE12, Efm 6123 are *vanB* type isolates.

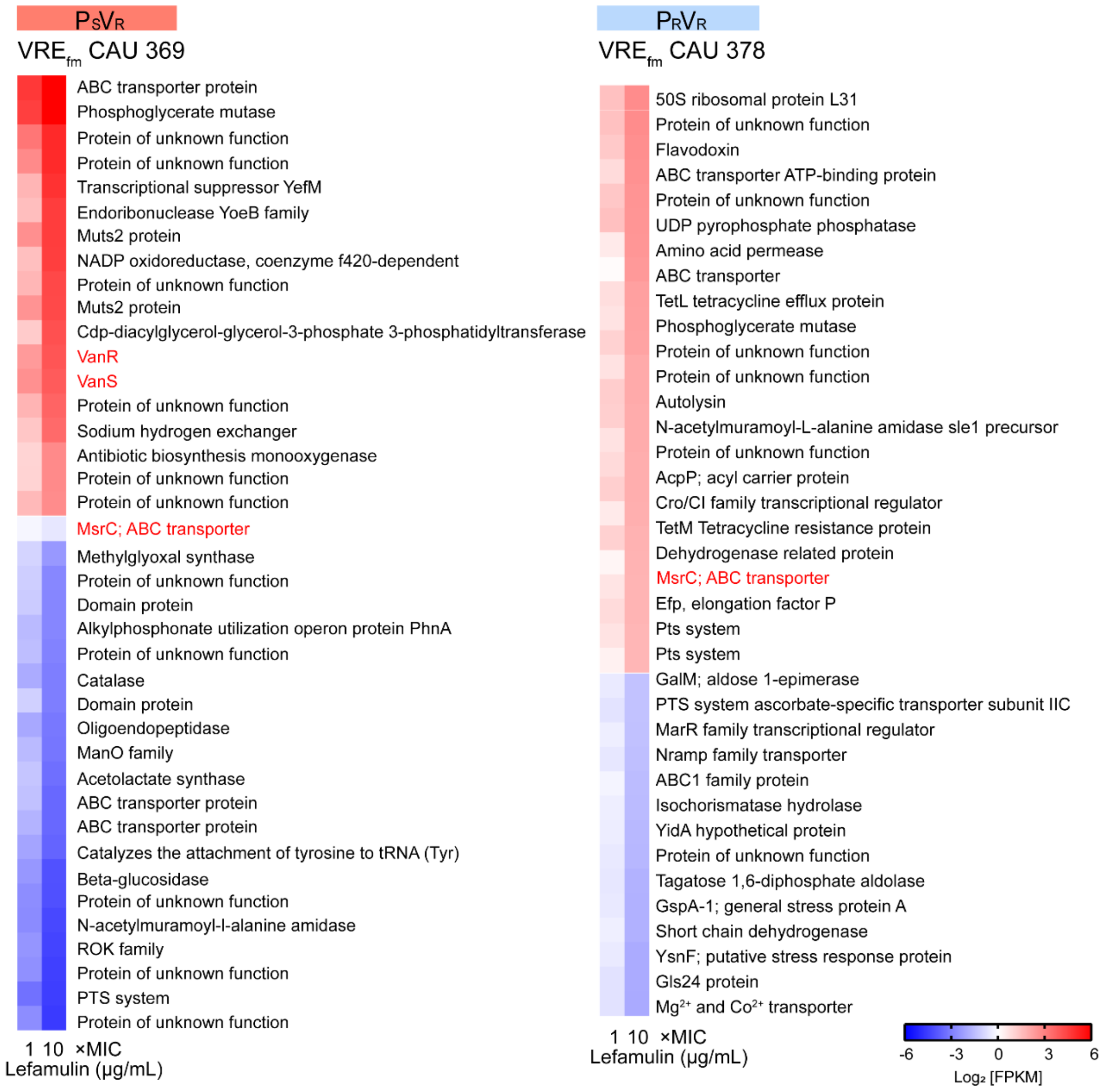

Supplementary Fig. 16 Activation of *vanRS* in P_sV_r VRE_{fm} with decreased *msrC* transcription.

Transcriptome analysis of VRE_{fm} CAU369 and CAU378 treated with 1× and 10×MIC lefamulin for 1 h. Genes were identified as significantly different with fold changes of $\log_2$ [FPKM] values of at fold increase or fold decrease at expression levels. The top 20 genes changed were shown from the original 2,960 genes.

Experiments were performed as three biologically independent experiments, and the mean ± S.D. (n = 3) is shown. *P* values were determined by non-parametric one-way ANOVA.

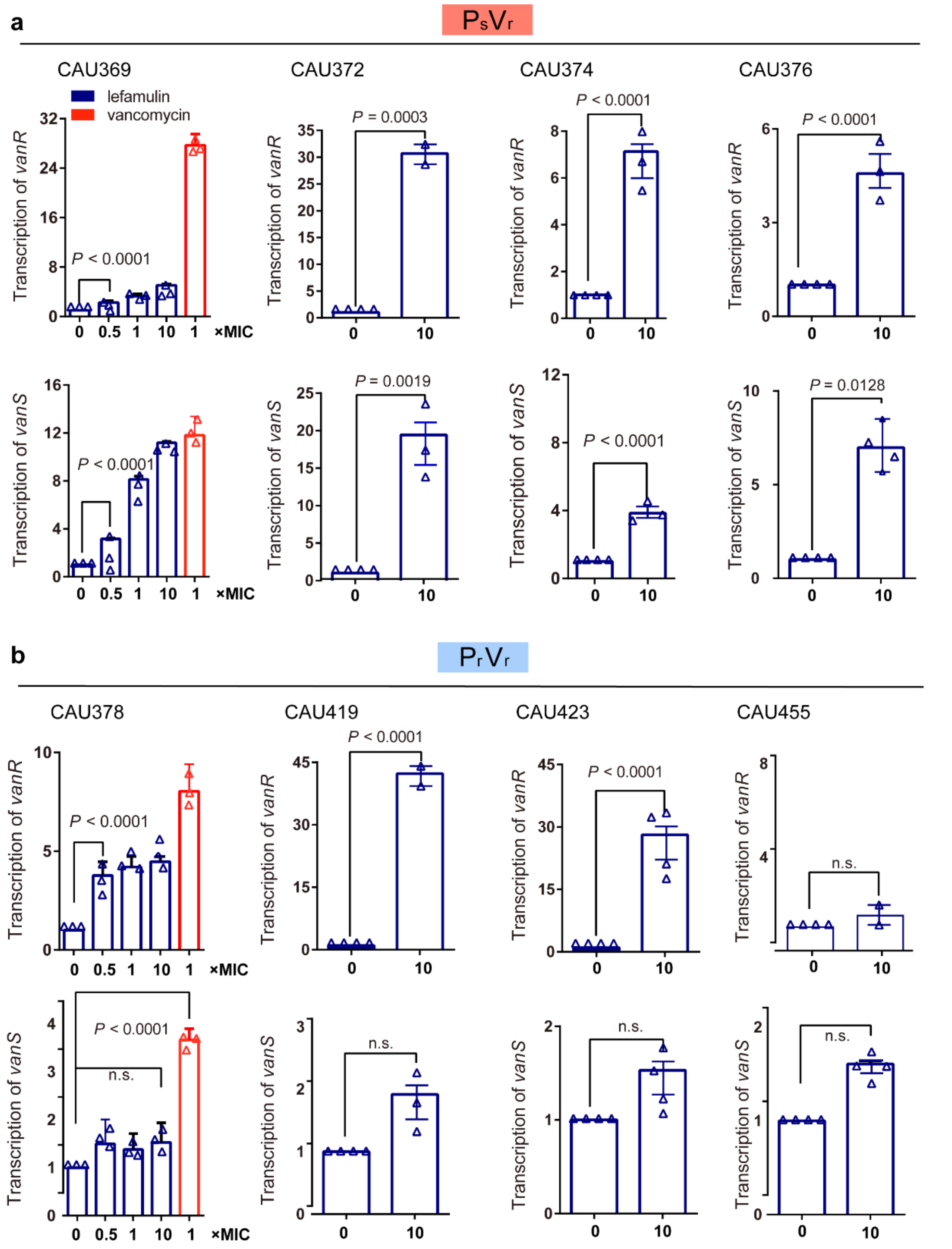

Supplementary Fig. 17 Lefamulin induces activation of *vanR* and *vanS* in P_sV_r isolates.

(**a-b**) Transcript ratios of the *vanR/vanS* were quantified in diverse P_sV_r (**a**) and P_rV_r (**b**) VRE_{fm} isolates based on qRT-PCR analysis.

Experiments were performed as three biologically independent experiments, and the mean ± S.D (n = 3). is shown. *P* values were determined by non-parametric one-way ANOVA.

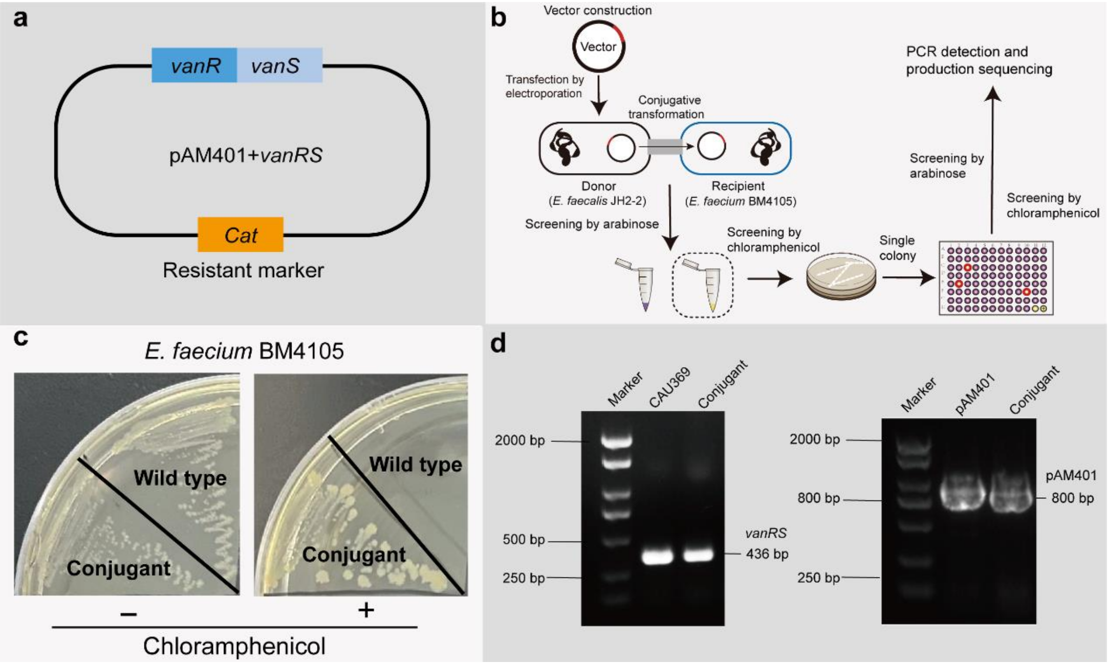

Supplementary Fig. 18 Vector construction and transfection scheme.

(**a**) Vector construction. Design of *vanRS* mutant plasmid**.**
(**b**) Transfection scheme of electroporation and conjugative transformation. Conjugant-2 (pAM401+*vanRS*) was screened through this pathway.
(**c**) Screening the conjugant carring pAM401+ *vanRS* plasmid in *E. faecium*. The conjugant shows chloramphenicol resistance.
(**d**) PCR amplification products of the conjugant. VRE$_{fm}$ CAU369 and pAM401 were used as controls. Experiments were performed as three biologically independent experiments

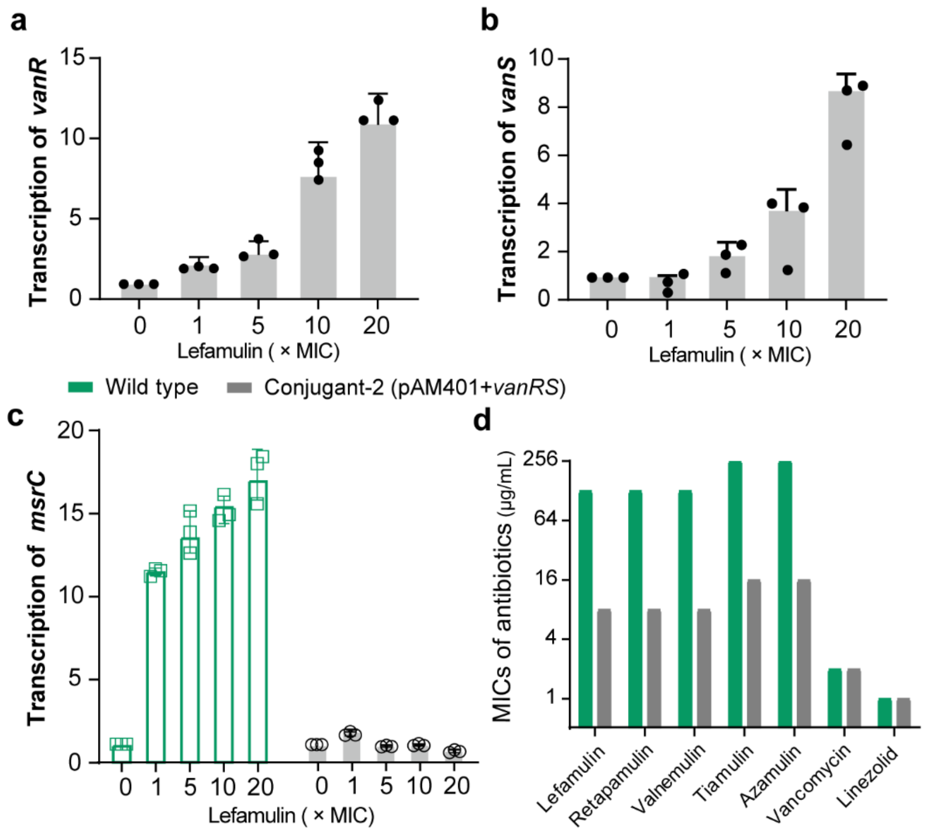

Supplementary Fig. 19 The *vanRS* inhibits the transcription of *msrC*.

(**a-b**) Transcription of *vanR* (**a**) and *vanS* (**b**) in the conjugant treated with lefamulin. (**c**) Comparsion of *msrC* transcription in wild type *E. faecium* and conjugant in the presence of lefamulin. Experiments were performed as three biologically independent experiments, and the mean ± S.D. (n = 3) is shown.

(**d**) Comparsion of the MICs of multiple antibiotics in wild type *E. faecium* and conjugant. Vancomycin and linezolid were used as controls.

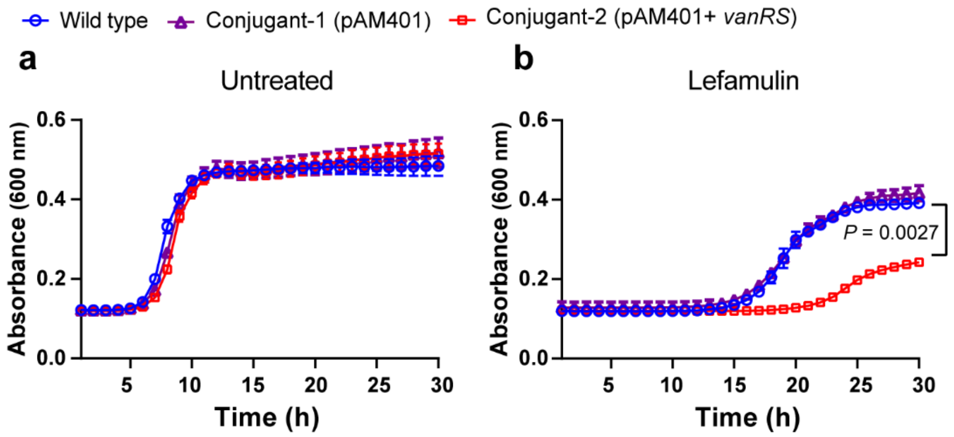

Supplementary Fig. 20 Lefamulin inhibits the growth of the conjugant carring *vanRS*.

Growth curves of wild type *E. faecium* and conjugants treated with a sublethal level of lefamulin (1/2 MIC). Conjugant-1 receives sole plasmid pAM401, whereas Conjugant-2 receives the plasmid pAM401+*vanRS*.
Experiments were performed as three biologically independent experiments, and the mean ± S.D. (n = 3) were shown. *P* values were determined by non-parametric one-way ANOVA.

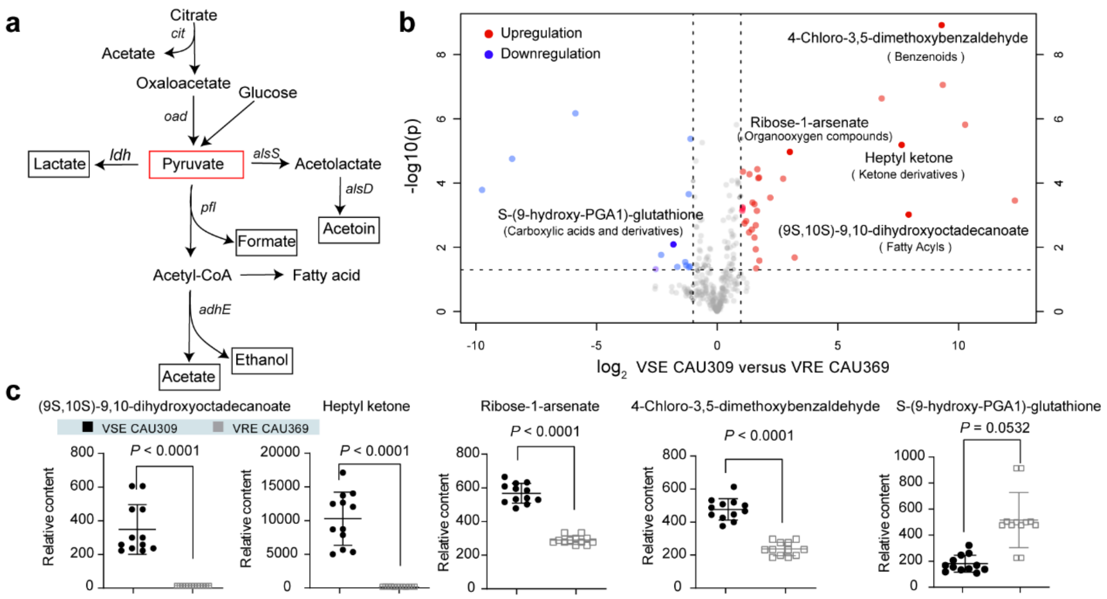

Supplementary Fig. 21 Pyruvate metabolism in *E. faeciums*.
(**a**) Scheme of pyruvate metabolism in *E. faeciums*, according to the reference[2]. Major end-products are boxed. Genes of *E. faeciums* encoding the synthesis of enzymes are shown as follows: *cit*, citrate lyase; *oad*, oxaloacetate decarboxylase; *ldh* L- (+) - lactate dehydrogenase; *alsS*, α-acetolactate synthase; *alsD*, α-acetolactate decarboxylase; *pfl*, pyruvate formate-lyase; *adhE*, aldehyde-alcohol dehydrogenase. (**b**) Volcano plot showing the metabolites in VRE_{fm} CAU369 and VSE_{fm} CAU309. (**c**) Comparison of five major metabolites in VRE_{fm} CAU369 and VSE_{fm} CAU309. All data are presented as mean ± S.D. (n = 12 biological replicates). *P* values were determined by non-parametric one-way ANOVA.

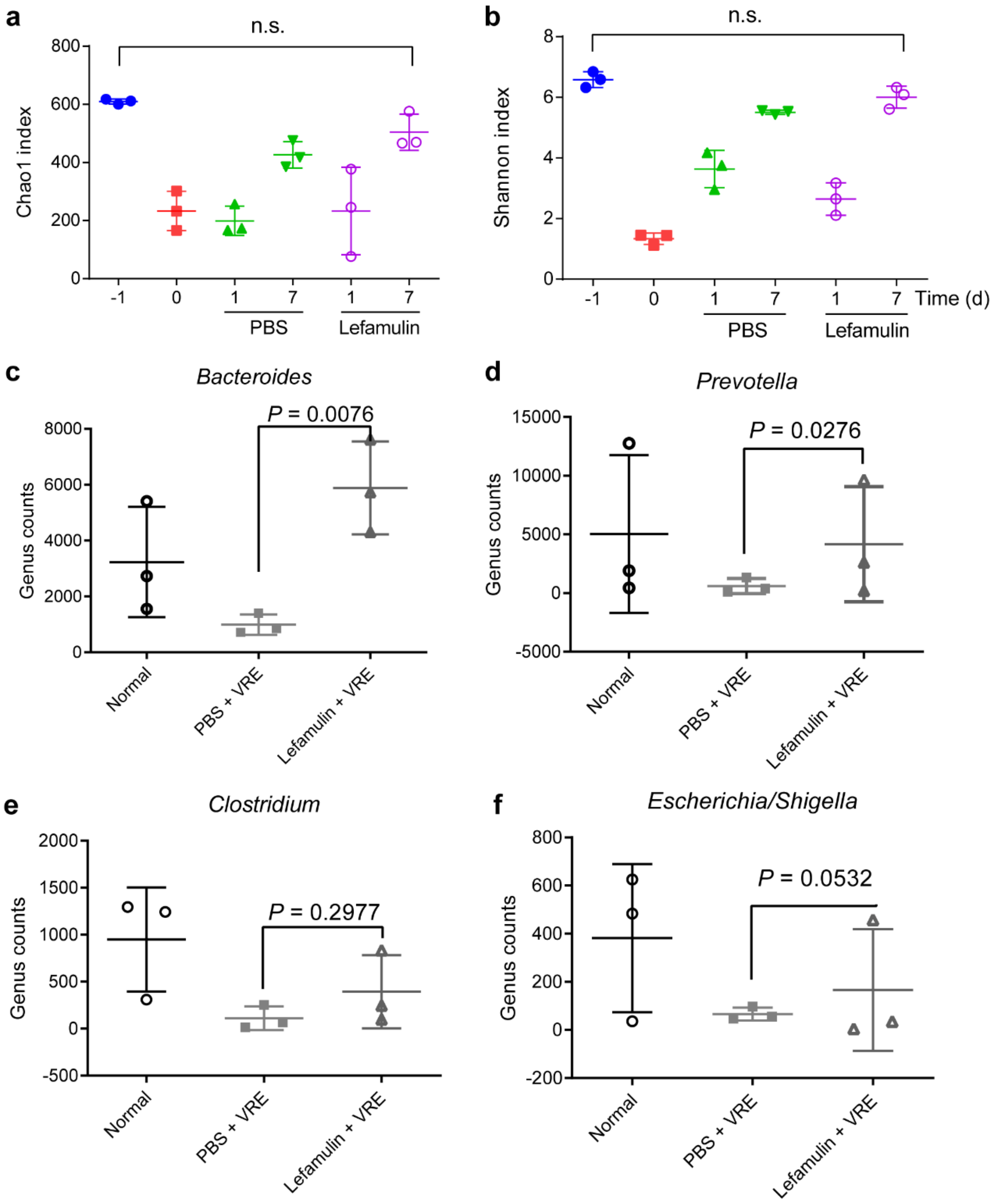

Supplementary Fig. 22 Lefamulin restores the homeostasis of intestinal flora.
(**a**-**b**) The Chao1 index (**a**) and Shannon index (**b**) of intestinal microbiota.
(**c**-**f**) Genus counts in the mouse intestine. *Bacteroides* (**c**) *Prevotella* (**d**) *Clostridium* (**e**) and *Escherichia/Shigella* (**f**) in the mice treated with lefamulin restored faster than that with PBS after expansion of VRE_{fm} CAU369 at the 7th day.
Experiments were performed as three biologically independent experiments, and the mean ± S.D. is shown. *P* values were determined by non-parametric one-way ANOVA.

Supplementary Table 1 Antibacterial activity and collateral sensitivity in *E. faecium* (n = 210).

Class	Antibacterial agent	MIC_{50} VRE (n = 101)	MIC_{50} VSE (n = 109)	RR	Target
Aminoglycoside	Gentamicin	>128	32	8	Ribosome, 30S
Amphenicol	Chloramphenicol*	32	8	4	Ribosome, 50S
β-lactam	Ampicillin	>128	2	64	PBP
	Cefoxitin	>128	64	2	
	Meropenem	>128	16	8	
Glycopeptide	Vancomycin	>128	2	64	D-Ala-D-Ala dipeptide
Lincosamide	Clindamycin	>128	4	16	Ribosome, 50S
Macrolide	Erythromycin	16	8	2	Ribosome, 50S
Oxazolidone	Linezolid	4	4	1	Ribosome, 50S
Pleuromutilin	Azamulin	0.5	>16	0.0312	Ribosome, 50S
	Lefamulin	0.06	>16	0.0037	
	Retapamulin	0.03	>16	0.0019	
	Tiamulin	0.5	>16	0.0312	
	Valnemulin	0.03	>16	0.0019	
Quinolone	Ciprofloxacin	>64	2	32	DNA gyrase
Rifamycin	Rifampicin	8	4	2	RNA polymerase
Streptogramin	Virginiamycin M1*	2	4	0.5	Ribosome, 50S
Tetracycline	Tetracycline	2	4	0.5	Ribosome, 30S

*Note: The MIC_{50} of chloramphenicol and virginiamycin M1 were calculated with 40 isolates.

Supplementary Table 2 MICs of multiple classes of antibiotics in 40 *E. faecium* (μg/mL)**.**

	Isolates	AMP	CIP	CLI	CRO	ERY	GEN	LZD	MEM	LMU	RIF	TET	VAN
	**CAU309**	128	128	128	128	128	128	4	128	16	16	16	2
	**CAU310**	128	128	64	128	128	128	2	128	16	8	1	2
	**CAU273**	1	0.5	0.5	64	8	16	2	8	16	16	0.5	2
	**CAU274**	2	2	4	128	4	16	4	16	16	16	1	2
	**CAU311**	128	128	128	128	128	128	4	128	0.125	4	16	1
	**CAU312**	4	4	0.25	128	128	32	2	16	0.03	16	16	1
	**CAU313**	2	2	128	128	128	16	4	16	16	4	16	1
	**CAU275**	4	2	4	64	8	16	4	16	16	8	0.5	2
	**CAU314**	128	128	128	128	128	128	4	128	16	16	16	2
**VSE_{fm}**	**CAU276**	8	16	32	128	16	8	4	16	16	0.25	4	2
	**CAU315**	128	128	128	128	128	128	2	128	0.125	4	16	2
	**CAU316**	4	16	128	128	128	128	4	16	16	0.25	4	1
	**CAU277**	4	0.3	0.25	128	8	0.25	0.25	16	0.03	0.25	1	2
	**CAU278**	2	2	4	128	4	16	4	16	16	4	0.5	1
	**CAU279**	4	4	64	128	32	32	4	32	16	16	0.25	2
	**CAU280**	2	1	8	8	16	16	2	16	16	32	2	1
	**CAU281**	0.25	1	1	4	2	32	1	0.25	16	4	0.25	1
	**CAU317**	2	1	64	128	16	2	4	16	16	64	4	1
	**CAU282**	4	16	32	128	32	8	4	32	16	16	0.25	2
	**CAU359**	>128	>64	>128	>128	>128	32	2	>128	0.06	16	0.5	> 128
	**CAU360**	>128	>64	>128	>128	>128	>128	2	>128	0.12	16	0.5	> 128
	**CAU187**	>128	>64	<0.25	>128	>128	16	2	>128	0.06	4	32	> 128
	**CAU361**	>128	>64	>128	>128	>128	8	2	>128	< 0.03	<0.25	<0.25	> 128
	**CAU362**	>128	>64	>128	>128	>128	>128	4	>128	0.03	8	1	> 128
	**CAU363**	>128	>64	<0.25	>128	<0.25	128	1	>128	< 0.03	1	<0.25	> 128
	**CAU364**	>128	>64	>128	>128	>128	>128	4	>128	2	4	0.5	> 128
	**CAU365**	>128	64	>128	>128	>128	>128	2	>128	2	2	0.5	> 128
	**CAU366**	>128	64	<0.25	>128	<0.25	>128	2	>128	0.25	4	32	> 128
	**CAU367**	>128	>64	<0.25	>128	<0.25	>128	2	>128	0.12	4	32	> 128
**VRE_{fm}**	**CAU368**	>128	>64	>128	16	>128	>128	2	<0.25	< 0.03	8	32	> 128
	**CAU369**	>128	>64	>128	>128	>128	>128	2	>128	< 0.03	4	2	> 128
	**CAU370**	>128	>64	<0.25	>128	8	16	2	>128	0.03	2	1	> 128
	**CAU371**	>128	>64	>128	>128	>128	16	2	>128	< 0.03	8	<0.25	> 128
	**CAU372**	>128	>64	>128	>128	>128	16	2	>128	< 0.03	4	32	> 128
	**CAU373**	>128	>64	>128	>128	>128	>128	2	>128	< 0.03	4	32	> 128
	**CAU374**	>128	>64	>128	>128	64	>128	1	>128	0.12	<0.125	<0.25	> 128
	**CAU375**	>128	>64	>128	>128	>128	>128	2	>128	0.03	4	32	> 128
	**CAU376**	>128	>64	>128	>128	>128	>128	1	>128	< 0.03	4	<0.25	> 128
	**CAU377**	>128	>64	>128	>128	>128	32	2	>128	0.12	4	16	> 128
	**CAU378**	>128	>64	>128	>128	>128	16	2	>128	> 16	4	32	> 128

**CAU996**	>128	>64	>128	>128	>128	8	1	>128	< 0.03	2	4	>128

Note: AMP: Ampicillin; CIP: Ciprofloxacin; CLI: Clindamycin; CRO: Ceftriaxone; ERY: Erythromycin; GEN: Gentamicin; LZD: Linezolid; MEM: RET: Retapamulin; RIF: Rifampicin; TET: Tetracycline; VAN: Vancomycin.

Supplementary Table 3 Information about VRE$_{fm}$ isolates worldwide.

	Accession ID (Bio sample)	Strain	Source
1	14694313	CAU187	China (This study)
2	14693177	CAU 359	
3	14693178	CAU 360	
4	14687682	CAU 361	
5	14693732	CAU 362	
6	14693733	CAU 363	
7	14693734	CAU 364	
8	14694296	CAU 365	
9	14694297	CAU 366	
10	14694298	CAU 367	
11	14694299	CAU 368	
12	14694301	CAU 369	
13	14694302	CAU 370	
14	14694303	CAU 371	
15	14694304	CAU 372	
16	14694306	CAU 373	
17	14694307	CAU 374	
18	14694308	CAU 375	
19	14694309	CAU 376	
20	14694310	CAU 377	
21	14694311	CAU 378	
22	3988550	ISMMS_VRE_2	America
23	3988560	ISMMS_VRE_5	
24	3988561	ISMMS_VRE_6	
25	3988626	ISMMS_VRE_8	
26	7274322	VRE2014-195	
27	7274321	VRE2014-7	
28	7274325	VRE2016-194	
29	7274324	VRE2016-78	
30	3198116	909_EFCM	
31	3197807	607_EFCM	
32	3197907	702_EFCM	
33	3197760	560_EFCM	
34	3197708	514_EFCM	
35	3197623	43_EFCM	
36	3197600	41_EFCM	
37	3197568	377_EFCM	
38	3197342	152_EFCM	
39	8595978	AUSMDU00004055	Australia
40	10273305	RBWH1	
41	4397503	805447/07	
42	6330395	LIM1590	Brazil
43	6555613	LIM695	
44	6555612	LIM559	
45	5461961	ERV196	Colombia
46	5461954	ERV157	
47	5461957	ERV175	
48	5461978	ERV98	
49	5461977	ERV35	
50	5461976	ERV34	
51	5461958	ERV177	Colombia
52	6621457	CFSAN059071	Denmark
53	6111320	GER_10_Efcm_HA-DE	Germany
54	6111319	9_Efcm_HA-DE	
55	6463381	2014-VREF-114	Korea
56	6472829	2014-VREF-63	
57	4500826	VREr7	Malaysia
58	4500822	VREr6	
59	6885147	VREr5	
60	4500812	VRE2	
61	5461973	MAL_ERV279	Mexico
62	5461972	Y_MAL_ERV275	
63	10248949	EF_386	Pakistan
64	10249139	EF_367	
65	10249219	EF_181	
66	10248944	PAK_EF_048	
67	10249011	PAK_EF_042	
68	10248976	PAK_EF_034	
69	10249190	EF_033	
70	10248976	EF_028	
71	10249104	EF_006	
72	4270932	SDW_VRE-1400294	Sweden
73	4270930	VRE-1300937	
74	4270929	VRE-1300911	
75	4270928	VRE-1300900	
76	4270927	VRE-1300899	
77	4270926	VRE-1300578	
78	4270925	VRE-1300518	
79	8196783	UK_VREF003	U.K.

Supplementary Table 4 Key nucleotides of 23S rRNA related to pleuromutilins resistance in 40 *E. faecium* isolates.

	Isolates	Copies of 23S rRNA	PLEs*	23S rRNA nucleotides							
				G2061	A2062	C2452	A2503	U2504	G2505	U2506	U2585
	**CAU273**	1	R	G	A	C	A	U	G	U	U
	**CAU274**	1	R	G	A	C	A	U	G	U	U
	**CAU275**	1	R	G	A	C	A	U	G	U	U
	**CAU276**	1	R	G	A	C	A	U	G	U	U
	**CAU277**	1	S	G	A	C	A	U	G	U	U
	**CAU278**	1	R	G	A	C	A	U	G	U	U
	**CAU279**	1	R	G	A	C	A	U	G	U	U
	**CAU280**	1	R	G	A	C	A	U	G	U	U
	**CAU281**	1	R	G	A	C	A	U	G	U	U
**VSE_{fm}**	**CAU282**	1	R	G	A	C	A	U	G	U	U
	**CAU309**	1	R	G	A	C	A	U	G	U	U
	**CAU310**	1	R	G	A	C	A	U	G	U	U
	**CAU311**	1	S	G	A	C	A	U	G	U	U
	**CAU312**	1	S	G	A	C	A	U	G	U	U
	**CAU313**	1	S	G	A	C	A	U	G	U	U
	**CAU314**	1	R	G	A	C	A	U	G	U	U
	**CAU315**	1	S	G	A	C	A	U	G	U	U
	**CAU316**	1	R	G	A	C	A	U	G	U	U
	**CAU317**	1	R	G	A	C	A	U	G	U	U
	**CAU187**	1	S	G	A	C	A	U	G	U	U
	**CAU359**	1	S	G	A	C	A	U	G	U	U
	**CAU360**	1	S	G	A	C	A	U	G	U	U
	**CAU361**	1	S	G	A	C	A	U	G	U	U
	**CAU362**	1	S	G	A	C	A	U	G	U	U
	**CAU363**	1	S	G	A	C	A	U	G	U	U
	**CAU364**	1	S	G	A	C	A	U	G	U	U
	**CAU365**	1	S	G	A	C	A	U	G	U	U
	**CAU366**	1	S	G	A	C	A	U	G	U	U
	**CAU367**	1	S	G	A	C	A	U	G	U	U
**VRE_{fm}**	**CAU368**	1	S	G	A	C	A	U	G	U	U
	**CAU369**	1	S	G	A	C	A	U	G	U	U
	**CAU370**	1	S	G	A	C	A	U	G	U	U
	**CAU371**	1	S	G	A	C	A	U	G	U	U
	**CAU372**	1	S	G	A	C	A	U	G	U	U
	**CAU373**	1	S	G	A	C	A	U	G	U	U
	**CAU374**	1	S	G	A	C	A	U	G	U	U
	**CAU375**	1	S	G	A	C	A	U	G	U	U
	**CAU376**	1	S	G	A	C	A	U	G	U	U
	**CAU377**	1	S	G	A	C	A	U	G	U	U
	**CAU378**	1	R	G	A	C	A	U	G	U	U

*Note: PLEs: Pleuromutilin antibiotics.

Supplementary Table 5 Information of 13 plasmids containing *vanA* genes in *E. faecium*

	Plasmids	Source	Length (bp)	Genbank	Reference
1	**pEMA120**		79797	KX853854.1	[3]
2	**pEM19081**	China	61320	NZ_KN880430.1	[4]
3	**pVRE1**		132733	CP040742.1	NCBI
4	**pZB18**	Japan	68058	AB611033.1	NCBI
5	**p2014-VREF-63**	Korea	287502	CP019989.1	NCBI
6	**pIP816**		34616	NC_011140.1	[5]
7	**pVEF3**		63135	AM931300	[6]
8	**pVEF1**	Norway	39626	NC_008768.1	[6]
9	**pVEF2**		39714	NC_008821.1	
10	**pVEF4**		44443	FN424376.1	NCBI
11	**pVRE001**		59226	CP018072.1	[7]
12	**pEFA-790c**	USA	35515	NZ_CP025755.1	NCBI
13	**pS177**		39032	HQ115078.1	[8]

Supplementary Tables 6 Primers for SELECT qPCR or qRT-PCR assay

SELECT qPCR primers	Sequences
23S-m6A1-up	TAGCCAGTACCGTAGTGCGTGTCAAACTACAGTAAAGCTCCATGGGGTCTT
23S-m6A1-down	TCCGTCCTGTCGCGGGTAACCTGCATCTTCCAGAGGCTGAGTCGCTGCAT
qPCR primers	Sequences
qPCR-F	ATGCAGCGACTCAGCCTCTG
qPCR-R	TAGCCAGTACCGTAGTGCGTG

Supplementary Tables 7 Primers for RT-PCR assay

Primers	Sequences	Reference
16S - F	CCTACGGGAGGCAGCAG	[9]
16S - R	ATTACCGCGGCTGCTGGC	
msrC - 1F	CAGCAAACTACGGACAAGCG	This study
msrC - 1R	GTCGGCGAAAATGGTTCAGG	
lsaE1 - F	AAGCCGAATGGTCTCGTTCC	This study
lsaE1 - R	CGCTGATCTGGGTCTCCATC	
eatAv - 1F	TTCAGGTCCTAACGGTGCAG	This study
eatAv - 1R	TCCATCCCAAGCTTTCGGAG	
vanS - F	CCGCTGCATACAGTGAGGAT	[10]
vanS - R	CCGTATCGGAAGAACGAGCA	
vanR - F	GGCACAAGCGGCCTTACTAT	
vanR - R	TAACTCCAGTGGGCGAAAGG	

Supplementary Table 8 MICs of multiple antibiotics in wild-type *E. faecium* and mutant (μg/mL).

	Pleuromutilin antibiotics							
	LMU	RET	VAL	TIA	AZA	VAN	LIN	ERY
Wild-type	0.06	0.06	0.06	0.25	0.25	2	1	1
Mutant (pAM401+*msrC*)	16	16	16	32	32	2	>128	>128

LMU: Lefamulin; RET: Retapamulin; VAL: Valnemulin; TIA: Tiamulin; AZA: Azamulin; VAN: Vancomycin; LIN: Lincomycin; ERY: Erythromycin.

References

1. Sun, L. Y. et al. Identification of novel conjugative plasmids with multiple copies of *fosB* that confer high-level fosfomycin resistance to Vancomycin-resistant *Enterococci*. *Front Microbiol* **8**, 1541 (2017).
2. Simons, J. A. Snoep, J. L. Feitz, S. Mattos, T. M. J. D. & Neijssel, O. M. Anaerobic 2-ketogluconate metabolism of Klebsiella pneumoniae NCTC 418 grown in chemostat culture: Involvement of the pentose phosphate pathway. *J Gen Microbiol,* **138**,423-428 (1992).
3. Schouten, M. A. et al. Vancomycin-resistant *Enterococci* isolated from patients in Europe shows geographic transposon type clustering. *Antimicrob Agents Chemother* 45, 986-989 (2001).
4. Arthur, M. Molinas, C. Depardieu, F. & Courvalin, P. Characterization of Tn1546, a Tn3-related transposon conferring glycopeptide resistance by synthesis of depsipeptide peptidoglycan precursors in *Enterococcus faecium* BM4147. *Bacteriol* **175**, 117-127 (1993).
5. Sletvold, H. et al. Complete sequence of *Enterococcus faecium* pVEF3 and the detection of an omega-epsilon-zeta toxin-antitoxin module and an ABC transporter. *Plasmid* **60**, 75-85 (2008)
6. Sletvold, H. et al. Comparative DNA analysis of two *vanA* plasmids from *Enterococcus faecium* strains isolated from poultry and a poultry farmer in Noway. *Antimicrob Agents Chemother* **51**, 736-739 (2007).
7. Honsa, E. S. et al. RelA mutant *Enterococcus faecium* with multi-antibiotic tolerance arising in an immunocompromised host. *mBio* **8**, e02124-02116 (2017).
8. Halvorsen, E. M. Williams, J. J. Bhimani, A. J. Billings, E. A. & Hergenrother, P. J. Txe, an endoribonuclease of the enterococcal Axe-Txe toxin-antitoxin system, cleaves mRNA and inhibits protein synthesis. *Microbiology* **157**, 387-397 (2010).
9. Lam, S. J. et al. Combating multidrug-resistant Gram-negative bacteria with structurally nanoengineered antimicrobial peptide polymers. *Nat. Microbiol*, 16162 (2016).
10. Silva, B. N. M. D. et al. Expression of *vanA*-type vancomycin resistance in a clinical isolate of *Enterococcus faecium* showing insertion of IS19 in the *vanS* gene. *Int J Antimicrob Agents* **55**, 105897 (2020).
