# Supplementary material for: Collateral sensitivity to pleuromutilins in vancomycin-resistant Enterococcus faecium
Source: Nat Commun. 2022 Apr 7;13:1888. doi: 10.1038/s41467-022-29493-0 (PMC8990069; doi:10.1038/s41467-022-29493-0)
Supplement: Supplementary file 2 — Reporting Summary [file 41467_2022_29493_MOESM2_ESM.pdf]

nature portfolio

Corresponding author(s): Kui Zhu

Last updated by author(s): Mar 10, 2022

Reporting Summary

Nature Portfolio wishes to improve the reproducibility of the work that we publish. This form provides structure for consistency and transparency in reporting. For further information on Nature Portfolio policies, see our Editorial Policies and the Editorial Policy Checklist.

Statistics

For all statistical analyses, confirm that the following items are present in the figure legend, table legend, main text, or Methods section.

n/a	Confirmed	
☐	☒	The exact sample size (*n*) for each experimental group/condition, given as a discrete number and unit of measurement
☐	☒	A statement on whether measurements were taken from distinct samples or whether the same sample was measured repeatedly
☐	☒	The statistical test(s) used AND whether they are one- or two-sided<br>*Only common tests should be described solely by name; describe more complex techniques in the Methods section.*
☐	☒	A description of all covariates tested
☐	☒	A description of any assumptions or corrections, such as tests of normality and adjustment for multiple comparisons
☐	☒	A full description of the statistical parameters including central tendency (e.g. means) or other basic estimates (e.g. regression coefficient) AND variation (e.g. standard deviation) or associated estimates of uncertainty (e.g. confidence intervals)
☐	☒	For null hypothesis testing, the test statistic (e.g. *F*, *t*, *r*) with confidence intervals, effect sizes, degrees of freedom and *P* value noted<br>*Give P values as exact values whenever suitable.*
☒	☐	For Bayesian analysis, information on the choice of priors and Markov chain Monte Carlo settings
☒	☐	For hierarchical and complex designs, identification of the appropriate level for tests and full reporting of outcomes
☒	☐	Estimates of effect sizes (e.g. Cohen's *d*, Pearson's *r*), indicating how they were calculated

Our web collection on statistics for biologists contains articles on many of the points above.

Software and code

Policy information about availability of computer code

Data collection	The OD600nm values, luminescence and fluorescence intensity were collected by Tecan Infinite M200 Microplate reader; The Ct values of RT-PCR were collected using ABI quantstudio™ 7 detection system.
Data analysis	GraphPad Prism 8 was used to plot graphs; Statistical analysis was performed by GraphPad Prism 8. Heatmap package of UHPLC-Q-Orbitrap were used to analyze the transcriptome data. MetaboAnalyst 4.0 were used to analyze metabolomics data.

For manuscripts utilizing custom algorithms or software that are central to the research but not yet described in published literature, software must be made available to editors and reviewers. We strongly encourage code deposition in a community repository (e.g. GitHub). See the Nature Portfolio guidelines for submitting code & software for further information.

Data

Policy information about availability of data

All manuscripts must include a data availability statement. This statement should provide the following information, where applicable:

- Accession codes, unique identifiers, or web links for publicly available datasets
- A description of any restrictions on data availability
- For clinical datasets or third party data, please ensure that the statement adheres to our policy

All data generated in this study are provided in the Supplementary Information/Source Data file. DNA and RNA-seq data are available in NCBI SRA with accession number PRJNA628015 [https://www.ncbi.nlm.nih.gov/bioproject/PRJNA628015]. The MS raw files and proteome sequences data used in this study have been deposited to in the Proteome Xchange Consortium under accession code PXD030906, but the data are available when manuscript online. The metabolomic data have been deposited to CNCB-NGDC under accession code PRJCA008528 [https://ngdc.cncb.ac.cn/bioproject/browse/PRJCA008528].

Field-specific reporting

Please select the one below that is the best fit for your research. If you are not sure, read the appropriate sections before making your selection.

☒ Life sciences ☐ Behavioural & social sciences ☐ Ecological, evolutionary & environmental sciences

For a reference copy of the document with all sections, see nature.com/documents/nr-reporting-summary-flat.pdf

Life sciences study design

All studies must disclose on these points even when the disclosure is negative.

Sample size	All experiments were performed with at least three independent biological replicates. Each value shown in figures was presented as the mean value of the biological replicates, with standard deviation shown as error bars.
Data exclusions	For differential expression analysis,the RNA expression levels for each gene with fragments per kilobase per million reads mapped (FPKM) lower than1 were removed from consideration before downstream analysis.
Replication	All of the reported results were reproducible in three independent biological.
Randomization	For animal studies, six to eight-week-old female ICR mice were randomly assigned to the experimental and the control groups (n=6 per group) by the experimenter.
Blinding	No blinding was required because all measurements are not subject to investigator's bias or ambiguity.

Reporting for specific materials, systems and methods

We require information from authors about some types of materials, experimental systems and methods used in many studies. Here, indicate whether each material, system or method listed is relevant to your study. If you are not sure if a list item applies to your research, read the appropriate section before selecting a response.

Materials & experimental systems

n/a	Involved in the study	
☒	☐	Antibodies
☒	☐	Eukaryotic cell lines
☒	☐	Palaeontology and archaeology
☐	☒	Animals and other organisms
☒	☐	Human research participants
☒	☐	Clinical data
☒	☐	Dual use research of concern

Methods

n/a	Involved in the study	
☒	☐	ChIP-seq
☒	☐	Flow cytometry
☒	☐	MRI-based neuroimaging

Animals and other organisms

Policy information about studies involving animals; ARRIVE guidelines recommended for reporting animal research

Laboratory animals	Six to eight-week-old female ICR mice.
Wild animals	None
Field-collected samples	None
Ethics oversight	The laboratory animal usage license number is SYXK-2016-0008, certified by Beijing Association for Science and Technology and the animal study protocols were performed in accordance with the relevant guidelines and regulations (ID: SKLAB-B-2010-003).

Note that full information on the approval of the study protocol must also be provided in the manuscript.
